# A platform for efficient identification of molecular phenotypes of brain-wide neural circuits

**DOI:** 10.1038/s41598-017-14360-6

**Published:** 2017-10-24

**Authors:** Tao Jiang, Ben Long, Hui Gong, Tonghui Xu, Xiangning Li, Zhuonan Duan, Anan Li, Lei Deng, Qiuyuan Zhong, Xue Peng, Jing Yuan

**Affiliations:** 1 Britton Chance Center for Biomedical Photonics, Wuhan National Laboratory for Optoelectronics-Huazhong University of Science and Technology, Wuhan, 430074 China; 2 MoE Key Laboratory for Biomedical Photonics, Collaborative Innovation Center for Biomedical Engineering, School of Engineering Sciences, Huazhong University of Science and Technology, Wuhan, 430074 China

## Abstract

A neural circuit is a structural-functional unit of achieving particular information transmission and processing, and have various inputs, outputs and molecular phenotypes. Systematic acquisition and comparative analysis of the molecular features of neural circuits are crucial to elucidating the operating mechanisms of brain function. However, no efficient, systematic approach is available for describing the molecular phenotypes of specific neural circuits at the whole brain scale. In this study, we developed a rapid whole-brain optical tomography method and devised an efficient approach to map brain-wide structural and molecular information in the same brain: rapidly imaging and sectioning the whole brain as well as automatically collecting all slices; conveniently selecting slices of interest through quick data browsing and then performing post hoc immunostaining of selected slices. Using this platform, we mapped the brain-wide distribution of inputs to motor, sensory and visual cortices and determined their molecular phenotypes in several subcortical regions. Our platform significantly enhances the efficiency of molecular phenotyping of neural circuits and provides access to automation and industrialization of cell type analyses for specific circuits.

## Introduction

A neural circuit is a structural-functional unit that achieves particular information transmission and processing^1^. The precise operation of specific neural circuits is a prerequisite for correct execution of brain function, whereas disorder in neural circuits is directly involved in the development of a variety of neurological diseases. Given that neural transmitters and functional proteins directly adjust and control neural circuits to perform specific functions^2^, the expression and distribution of important molecules are important for identifying neuron types and related circuit features. Therefore, dissecting global wiring properties and integrating molecular features in neural circuits are vital to understanding how neurons in the circuits receive, process and relay information^3^.

However, systematically identifying molecular features in neural circuits is challenging. The diversity of neural functional molecules leads to broad variability in neural molecular phenotypes. Furthermore, neural circuits involved in special brain functions usually require a whole-brain scale^4^. Therefore, elucidating the structure-function relationships in a neural circuit requires comprehensive anatomical and molecular phenotypical maps at the cellular level across a brain-wide scale. Unfortunately, it is a labour-intensive and time-consuming process to use conventional manual methods to characterize diverse anatomical and molecular traits that span a large region of the brain.

Recently, chemical clearing^5–14^, coupling with light-sheet microscopy^8,15–18^, has made substantial progress to characterize molecular phenotypes of whole-mount tissue. Replacing or removing lipids increases the tissue porosity for transparency, which facilitates the penetration of antibody molecules into deep tissue and enables whole-mount immunostaining. However, the complexity and slow nature of whole-mount immunostaining protocols lead to quenching of the staining signals. Additionally, it is important to consider cost efficiency owing to consuming large doses of antibodies. More importantly, although chemical clearing is compatible with small-molecule antibodies, the penetration depth and uniformity of antibodies in thick tissue may be reduced through the increase in the molecular size of antibodies. Moreover, it is hard to map and phenotype the cleared whole brain with high resolution owing to technical limitations of light-sheet microscopy^19^. Therefore, there is a particularly urgent need for a novel, cheap and efficient method to acquire massive amounts of data pertaining to the molecular phenotypes of neural circuits in imaging at the whole-brain scale with single-cell resolution.

In this study, we devised an alternative approach to identify molecular phenotypes in neural circuits at the whole-brain scale with high efficiency. Our approach consists of the following three steps: automated imaging and sectioning of the whole brain and collecting all slices sequentially in parallel; rapid browsing of the whole dataset and selecting specific slices with anatomical information of specific neural circuits; and then performing post hoc immunostaining on these selected slices for classifying the molecular phenotypes of special neural populations. For this pipeline, we developed a whole-brain tomography system capable of rapid whole-brain imaging and automatic slice collection. Using this platform, we demonstrated the diverse patterns of inputs to motor, sensory and visual cortices. We also quantitatively analysed and compared molecular features of the inputs across several subcortical regions. Our system has the potential to be widely applied as a routine platform with high efficiency to systematically determine detailed molecular characteristics of neural circuits.

## Results

### Pipeline for phenotyping brain-wide neural circuits

**Figure 1:**
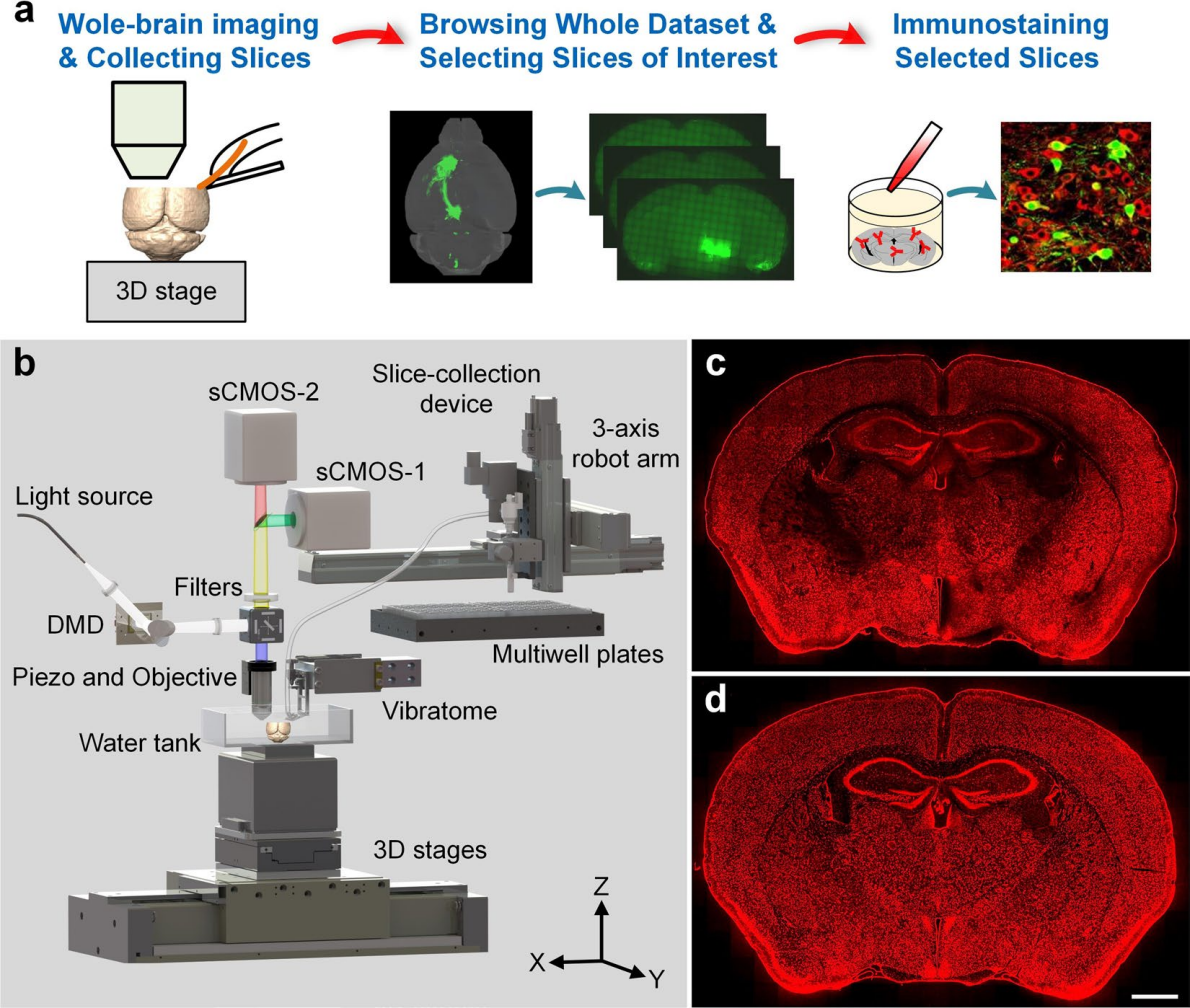
Phenotyping brain-wide neural circuits. (**a**) Presentation of the whole pipeline. (**b**) Schematic of the rapid whole-brain optical tomography system with automatic slice collection. (**c**) and (**d**) Typical coronal images of propidium iodide (PI)-stained 2 M C57BL/6 J mouse brains acquired by the system before and after optimization. Scale bar: 1 mm.

We built a pipeline for identifying the molecular phenotypes of brain-wide neural circuits, which included three steps, as shown in Fig. 1a. The first step was to acquire a whole-brain dataset by rapid whole-brain optical imaging and to collect all of the imaged slices simultaneously. For this step, we employed a combination of vibrating sectioning and wide-field imaging to enhance the data acquisition speed. Moreover, we designed a water-flow device to achieve slice collection. The second step was to browse the self-registered whole dataset and select slices of interest immediately after data acquisition. The last step was to adopt a traditional approach to perform subsequent immunostaining on the selected slices. The first two steps significantly improved the efficiency of molecular phenotyping of brain-wide neural circuits by replacing the manual steps of sectioning, pasting and imaging individual slices in traditional histology.

To scan the brain-wide projection patterns of neural circuits quickly and save all serial slices automatically for post hoc staining, we constructed a rapid whole-brain optical tomography with automatic slice collection. This system consists of wide-field imaging, vibrating sectioning and slice collection (Fig. 1b and Supplementary Fig. 1). A structured illumination microscope (SIM)^20^ was employed to provide wide-field optical-sectioning imaging of an agarose-embedded sample. Owing to the high-speed binary modulation and adjustable pattern period of digital mirror device (DMD), the system enabled high-throughput optical sectioning with a flexible optical sectioning thickness (Supplementary Fig. 2). In this study, because the size of the neuron body is generally on the order of 10 microns, we chose an axial response of 5.8 µm at 510 nm to balance the effective information and signal-to-noise ratio in whole-brain imaging. Lateral resolution was 0.32 × 0.32 µm in the following whole-brain imaging experiments. A homemade high-precision vibratome (Supplementary Fig. 3a) was integrated into the system to section imaged tissue into entire slices. We employed energized rectangular coils in a magnetic field to actuate a double-leafspring vibrating structure. This design eliminated the possibility of mechanical wear and improved the prolonged stability of the system. We also designed an adjustable frame to adjust the rolling angle of the blade in high precision to minimize Z displacement of the blade. A three-dimensional (3D) stage accurately moved the sample mosaic-by-mosaic to extend the field of view (FOV) in the imaging part, and then the stage moved the sample towards the oscillatory blade to generate slices in the sectioning part. The slice thickness can be flexibly selected as needed, but the minimum slice thickness for interval-sampling imaging is 40 µm to ensure the integrity of the slices for subsequent post-hoc immunolabelling. We also established a water-recirculating device to transfer the slices into customized multi-well plates. The cycle of imaging, sectioning and collecting (Supplementary Movie 1) was repeated until the whole-brain dataset acquisition was finished and all the slices were collected sequentially for post hoc staining. The slice-collection process of 30 s was so quick that the system could collect current slices during imaging of the next section. Collecting the slices had no influence on the total data acquisition time of whole-brain imaging.

After optimization, the Z displacement of the blade was controlled within 2.5 µm at the vibration amplitude of 0.5 to 3 mm (Supplementary Fig. 3c). And the root mean square (RMS) variation of the sectioned agarose surface was 0.4 ± 0.1 µm in X and 0.8 ± 0.1 µm in Y (mean ± SD; sample number = 5, n = 3 for each sample; Supplementary Fig. 3d and e). Furthermore, we employed optical-sectioning imaging of the real time-stained^21^ superficial tissue (Supplementary Fig. 4) to evaluate the sectioning quality of agarose-embedded C57BL/6 J mouse brain tissue. Before system optimization, poor sectioning quality led to slight and even total signal losses in partial regions (Fig. 1c and Supplementary Fig. 5a). As a comparison, Fig. 1d and Supplementary Fig. 5b show serial sections with an entire uniform signal distribution, illustrating the adequate flatness of the sectioned tissue. These results demonstrated that our system was capable of brain-tissue sectioning with excellent performance.

In addition to interval-sampling imaging, our system was also capable of acquiring a full-volume dataset of the whole brain. To demonstrate this ability, we imaged a 2 M *Thy1*-eGFP M-line mouse^22^ brain (Supplementary Fig. 6a). Owing to the transmission depth limitation of the modulated patterns, we acquired 2 optical sections axially at z-steps of 5 µm and then sectioned the imaged tissue at a thickness of 10 μm. In this case, no intact sample slices were generated for subsequent post-hoc immunolabelling. The whole-brain dataset of 2,870 coronal sections was collected in 72 hours, including approximately ~56 hours for imaging and ~16 hours for sectioning. The morphology of GFP-labelled cortical pyramidal neurons was clearly resolved in Supplementary Fig. 6b–d, including the cell somas, dendrites and axons. A 3D volume rendering of local brain regions is shown in Supplementary Fig. 6e, including the somatosensory cortex, auditory cortex, part of the hippocampus and thalamus. The orientation of axon fibres and the distribution of neurons were clearly visualized. The results showed that our system was capable of automated acquisition of a high-resolution full-volume dataset of fluorescence-labelled mouse brain.

### Automatic slice collection during whole-brain imaging

**Figure 2:**
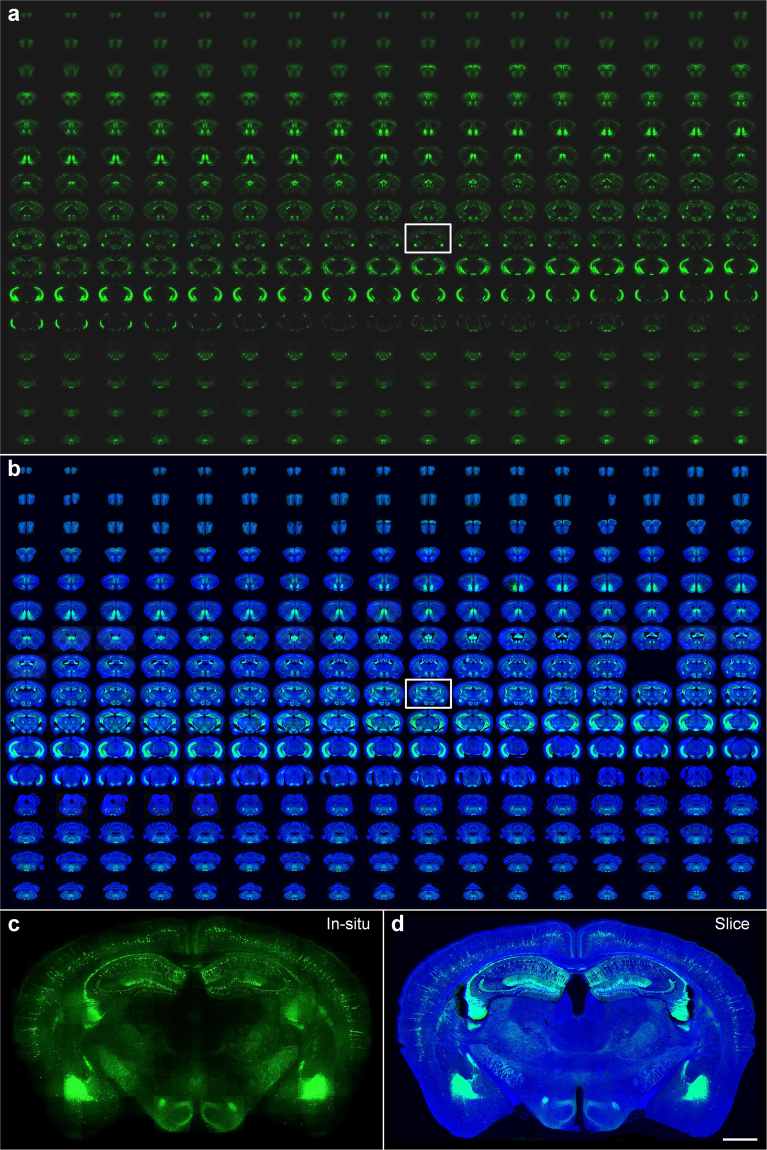
Automatic slice collection of imaging a *Thy1*-eYFP H-line mouse brain. (**a**) The brain was imaged by our system as 272 coronal sections at an interval of 50 μm, and all imaged tissue slices were sequentially collected during data acquisition. (**b**) All collected slices were placed on the slides and imaged with a Nikon Eclipse Ni-E wide-field microscope one-by-one manually. Blue and green represent 4′, 6-diamidino-2-phenylindole (DAPI) staining and YFP-labelled images of the collected slices, respectively. All images are arranged according to the corresponding *in situ* images, and the blank areas represent the missing slices. Enlarged views of the hippocampal coronal plane are indicated by white rectangles in row 9 and column 10. (**c**) An *in situ* image during whole-brain imaging with our system, and (**d**) the image of the corresponding collected slice imaged with a Zeiss LSM 710 confocal microscope. Scale bars in (**c**) and (**d**) are 1 mm.

To validate the automatic sequential collection of the imaged slices, we imaged a 2 M *Thy1*-eYFP H-line mouse^22^ brain and collected all serial slices simultaneously. A whole-brain dataset of 272 sections at the sectioning thickness of 50 μm was acquired in 11.0 hours (Fig. 2a), which demonstrated the capability of our system to conduct rapid imaging of a whole brain. Figure 2b shows the images of all the collected slices, from the olfactory bulb to the cerebellum, using a commercial microscope with a manual approach. Overall 266 intact slices were automatically saved, 2 slices were missed, 4 slices were partially lost, and the success rate of the slice collection was 97.8%. The partial-loss slices shown in Fig. 2b were located in the olfactory bulb, midbrain and cerebellum. Tissue around these brain regions was separated naturally. Compared with the corresponding *in situ* images before sectioning shown in Fig. 2a, the vast majority of the collected slices were morphologically intact (Fig. 2b). Figure 2c and d also show the enlarged morphological comparison of the *in situ* image in the hippocampus during whole-brain imaging and the image of the corresponding collected slice. The slice integrity benefited from transferring the slices by soft water flow in a silicon pipe. This special design, which avoided contacting the mechanical parts of the pinch valves and the pump, eliminated potential damage. We performed this experiment on 3 other mouse brains, and the average success rate of slice collections was 97.4% (Supplementary Table 1). We further carried out the slice collection experiments of 3 mouse brains at a slice thicknesses of 40 μm, and the average success rate of the slice collections still reached 96.7%. An improved crosslinking strength between agarose and brain tissue may potentially avoid the partial loss of slices and improve the success rate further. These results indicated that our system was capable of not only revealing brain-wide anatomical distribution for rapid whole-brain data browsing but also gathering all of the imaged slices without damage for potential subsequent molecular identification.

### Rapid whole-brain imaging for selective post hoc immunostaining of automatically collected slices

**Figure 3:**
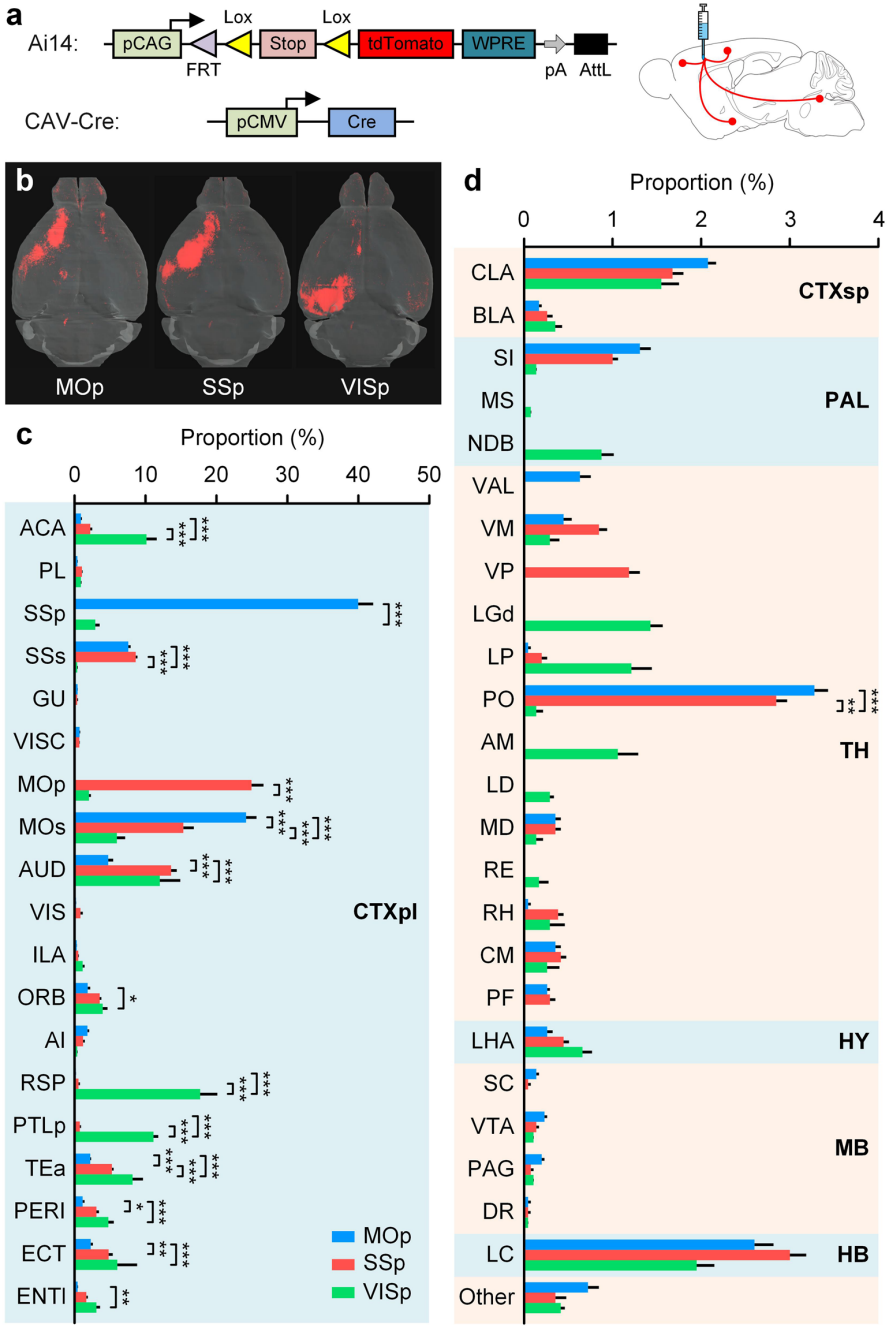
Whole-brain mapping of inputs to MOp, SSp and VISp. (**a**) Schematic of direct inputs to targeted areas using stereotaxic injections of *CAV-Cre*. Left side shows the gene elements of the *Ai14* Cre-reporter mouse and *CAV-Cre* used in a labelling experiment. Right side shows that *CAV-Cre* was injected into the targeted area of a mouse brain. (**b**) Whole-brain volume rendering of the inputs to MOp, SSp and VISp. (**c**,**d**) Quantification of the brain-wide inputs in cortical and subcortical brain areas to MOp, SSp and VISp. Error bars represent SEM. Others represent all other input regions. The significant differences between pairs are indicated by the p value (*p < 0.05, **p < 0.01, and ***p < 0.001). Injection sites were excluded from the data analysis.

To demonstrate the capability of our system to acquire whole-brain anatomical and slice-of-interest molecular information, we systematically mapped whole-brain inputs to the primary motor area (MOp), primary somatosensory area (SSp) and primary visual area (VISp) and identified the molecular phenotypes of these inputs in specific brain regions. We performed whole-brain imaging on adult (3–6 months old) *Ai14* Cre-reporter mice^23^ (Stock number: 007908, Jackson Laboratory) injected with the retrograde canine adenovirus type 2-Cre (CAV-Cre)^24,25^ virus in MOp (n = 6), SSp (n = 6) and VISp (n = 5) (Fig. 3a). For each mouse, the whole-brain dataset included approximately 360 equidistant coronal sections that were obtained at a sectioning thickness of 40 µm in approximately 13.5 hours, and almost all of the imaged slices were automatically collected. Self-registration of the datasets enabled us to conveniently visualize 3D signal distributions over the whole brain from sequential two-dimensional (2D) images. Thus, all of the datasets were examined immediately to evaluate the accuracy of virus injection and the effects of fluorescence expression once the data acquisition was completed. A typical 3D volume rendering of these three kinds of whole-brain datasets illustrated the diversity of input patterns, as shown in Fig. 3b. High resolution enabled us to clearly resolve fluorescence-labelled somas (Supplementary Figs 7–9). Coronal images were manually registered to the Allen atlas^26^ and then anatomical structures were identified (Table 1). We examined the datasets and figured out that specific cortical and subcortical brain regions projecting to MOp, SSp and VISp.

**Table 1:** Abbreviations for anatomical structures.

Abbreviation	Definition	Abbreviation	Definition
**CTXpl**	Cortical plate	**TH**	Thalamus
ACA	Anterior cingulate area	VAL	Ventral anterior-lateral complex
PL	Prelimbic area	VM	Ventral medial nucleus
SSp	Primary somatosensory area	VP	Ventral posterior complex
SSs	Supplemental somatosensory area	LGd	Dorsal part of the lateral geniculatecomplex
GU	Gustatory areas	LP	Lateral posterior nucleus
VISC	Visceral area	PO	Posterior complex
Mop	Primary motor area	AM	Anteromedial nucleus
MOs	Secondary motor area	LD	Lateral dorsal nucleus
AUD	Auditory areas	MD	Mediodorsal nucleus
VIS	Visual areas	RE	Nucleus of reunions
ILA	Infralimbic area	RH	Rhomboid nucleus
ORB	Orbital area	CM	Central medial nucleus
AI	Agranular insular area	PF	Parafascicular nucleus
RSP	Retrosplenial area	**HY**	Hypothalamus
PTLp	Posterior parietal association areas	LHA	Lateral hypothalamic area
Tea	Temporal association areas	**MB**	Midbrain
PERI	Perirhinal area	SC	Superior colliculus
ECT	Ectorhinal area	VTA	Ventral tegmental area
ENTl	Entorhinal area, lateral part	PAG	Periaqueductal gray
**CTXsp**	Cortical subplate	DR	Dorsal nucleus raphe
CLA	Claustrum	**HB**	Hindbrain
BLA	Basolateral amygdalar nucleus	LC	Locus ceruleus
**PAL**	Pallidum		
SI	Substantia innominata		
MS	Medial septal nucleus		
NDB	Diagonal band nucleus		

We manually counted the input neurons to MOp, SSp or VISp in all input regions and then quantified their proportion of the total input neurons within each mouse brain (Fig. 3c,d). The significance within each input region was tested by two-way analysis of variance (ANOVA) and a post hoc Tukey’s multiple comparison test. We detected over 3,408 ± 338 (Mean ± SEM) clearly labelled neurons to MOp, 8,025 ± 1,046 inputs to SSp and 5,955 ± 1,221 inputs to VISp.

For all three modalities, the majority of the external input neurons were located in the cortex (MOp: 89.2% ± 0.3%, SSp: 88.6% ± 0.3% and VISp: 90.6% ± 0.6%). All of the MOp, SSp and VISp received many inputs from the same regions in the cortex, including ACA, MOs, AUD, ORB, TEa, PERI, ECT and CLA, which was consistent with previous studies^27,28^. Some of these common inputs showed different projection intensities for these three targeted regions. We found more extensive inputs from AUD to SSp and VISp than those to MOp. We also found that the inputs to MOp and SSp were more similar among these three targeted regions. Compared to VISp, MOp and SSp received a significantly lower proportion of the inputs from ACA and a significantly higher proportion of the inputs from SSs. These results supported that MOp and SSp involved in somatic sensorimotor networks^27,29^. Furthermore, RSP and PTLp were densely connected only with VISp, which suggested that they mainly belong to the visual network, which is similar to the results of previous studies^27,29^. Three brain regions received various proportions of the inputs from MOs, and TEa.

Subcortical regions constituted the minority of these three inputs. All of the MOp, SSp and VISp cortices received some similar inputs, such as LC^30,31^, VM, CM and LHA. MOp and SSp preferentially received the inputs from PO, MD and PF in the thalamus^32^ and SI in PAL, which further indicated the similarity of MOp and SSp. RH in the thalamus contributed to the higher proportion of the inputs to both SSp and VISp compared to MOp. No data demonstrated that any brain region provided a higher proportion of the inputs to both MOp and VISp compared to SSp. In addition, some brain regions only projected to MOp, SSp or VISp. In the thalamus, VAL projected to MOp, VP connected to SSp, and AM, LD, LGd accessed to VISp, which was consistent with previous studies^28,32^. Similarly, NDB in PAL only jointed with VISp^33,34^.

**Figure 4:**
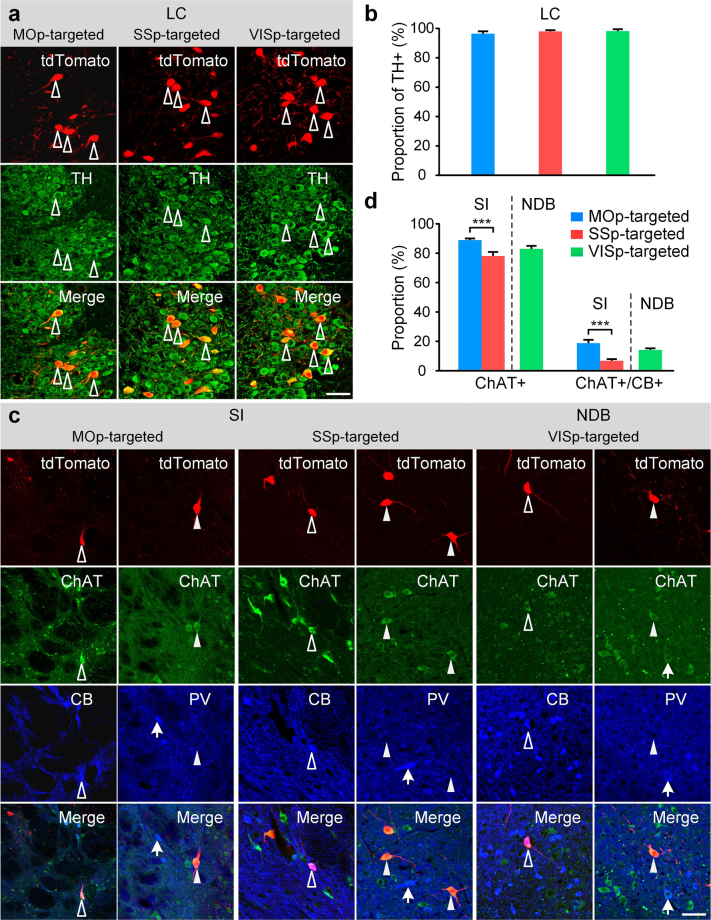
Characterizing the molecular phenotypes of input neurons projecting to MOp, SSp and VISp in LC and PAL. (**a**) tdTomato-labelled LC slices with TH immunostaining. The hollow arrowheads show the co-localization of tdTomato-labelled input neurons and TH+ neurons. (**b**) The percentage of tdTomato-labelled input neurons in LC that express TH. (**c**) tdTomato-labelled PAL slices with ChAT and CB or ChAT and PV dual immunostaining. Hollow arrowheads show tdTomato-labelled input neurons co-localizing with ChAT +/CB+; solid arrowheads indicate the co-localization of tdTomato-labelled input neurons and ChAT+/PV- neurons; and arrows show the PV + neurons without tdTomato-labelled co-localization. (**d**) The percentage of tdTomato-labelled input neurons in the subregions of PAL: MOp-targeted and SSp-targeted input neurons in SI, and VISp-targeted input neurons in NDB. Data represent the mean ± SEM. The significant differences between pairs (MOp-targeted vs. SSp-targeted in SI) are indicated by the p value (Unpaired two-tail t-tests, *p < 0.05, **p < 0.01, and ***p < 0.001). Scale bars: 50 μm. Blue, red and green represent MOp-targeted, SSp-targeted and VISp-targeted neurons, respectively.

As mentioned above, some brain regions showed connections with all of the targeted regions, whereas the input distributions of some regions to these three targeted regions had distinct region specificities. We selected LC and PAL as regions of interest and identified the molecular phenotypes of the input neurons to MOp, SSp and VISp in the two regions (Fig. 4). We performed post hoc immunostaining of the corresponding collected slices from these mice.

We immunostained all the slices corresponding to LC area with a tyrosine hydroxylase (TH, an enzyme in the biosynthetic pathway for noradrenaline) antibody.The images of fluorescence-labelled and immunostained slices showed that almost all of the tdTomato-labelled input neurons were TH-positive (TH+) (Fig. 4a). Overall, 96.0 ± 1.0% of input neurons of MOp (total 721/749) were TH+, 97.5 ± 0.4% of input neurons of SSp (total 2325/2388) were TH+, and 97.7 ± 0.7% of input neurons of VISp (total 1010/1029) were TH+ (Fig. 4b).

As mentioned above, three targeted regions received the inputs from different subregions of PAL. We further identified the main molecular features of the inputs in SI and NDB, which belong to neuromodulatory subregions of PAL. We selected antibodies against choline acetyltransferase (ChAT), calbindin-1 (CB, calcium-binding protein) and parvalbumin (PV) to stain the inputs to MOp and SSp in SI and the inputs to VISp in NDB (Fig. 4c). The images of fluorescent-labelled and immunostained slices showed that most of the tdTomato-labelled input neurons connecting to MOp or SSp were ChAT-positive (ChAT+, 88.3 ± 0.6% for MOp, 77.5 ± 2.3% for SSp, 82.3 ± 1.5% for VISp) (Fig. 4d), which was consistent with previous studies^35,36^. A small portion of input neurons co-expressed ChAT and CB (18.2 ± 1.6% for MOp, 6.0 ± 0.6% for SSp, 13.6 ± 0.6% for VISp), which was similar to previous studies^37^. No data showed PV-positive (PV+) neurons in PAL projected to MOp, SSp or VISp. These results demonstrated that the subregions in PAL had preferences for projection to these targeted regions, and the input neurons in the subregions had similarities among main molecular features with different proportions.

## Discussion

With viral and genetic labelling techniques, whole-brain imaging technologies have allowed researchers to trace and reconstruct specific neural circuits to map the global wiring of neural networks^28^. Various neurons that constitute neural circuits have different inputs, outputs and molecular phenotypes. To further our understanding of how neurons process information from input sites and relay information to their output sites, the mapping and phenotyping of specific neural circuits in the whole brain is an area of intense research focus. For this objective, we proposed an efficient pipeline to integrate neuron anatomy with molecular signatures to achieve multi-dimensional identification of neural circuits. Our method replaces the manual steps of sectioning, pasting and imaging individual slices with traditional histology^27^. Our approach gives researchers a tool to browse the whole brain and identify regions of interest quickly and conveniently. This approach saves labour and significantly enhances efficiency as well as greatly improves the throughput of data collection. Furthermore, effortless whole-brain reconstruction benefiting from self-registration can provide a visual and precise 3D distribution of brain-wide neural circuits. Compared to previous automatic whole-brain optical imaging technologies^21,38–40^, our method can collect almost all imaged slices and allow researchers to freely select slices of interest and perform subsequent immunostaining or other experiments (such as *in situ* hybridization). In this study, we systematically examined the brain-wide inputs of MOp, SSp and VISp, and we further identified the main phenotypes of neuromodulatory input regions. We observed that both the motor and sensory cortex as well as the visual cortex had different inputs from subregions of PAL but similar molecular phenotypes. Of course, it is important to consider potential caveat regarding the tropism of CAV, even though CAV has shown to infect many classes of neurons, such as motor, cholinergic and dopamine neurons^24^. Another potential caveat regarding infection efficiency is that CAV permits only modest levels of transgene expression^41^.

Our whole-brain tomography system achieved rapid whole-brain imaging and automatic slice collection, and provided a relatively easy and cost-effective solution for the molecular identification of specific neural circuits. Although this system has the ability to provide full volumetric imaging, the vibrating sectioning allowed us to shorten the whole-brain imaging time by interval sampling. Differing in two-photon excitation imaging in serial two-photon tomography (STP)^40^, we adopted structural illumination microscopy to considerably enhance the imaging throughput. To acquire massive data for brain connectivity, long-time running of Ti:sapphire lasers in STP undoubtedly led to high costs in device purchase and maintenance. In addition, combining the broad spectrum of the mercury arc lamp with appropriate filters can easily provide multi-excitation light. Compared with two-photon excitation, this approach had a potential benefit of simultaneously imaging multi-colour samples due to narrower excitation spectrum of one-photon excitation. Though the out-of-focus excitation of the wide-field illumination led to potential photobleaching, interval-sampling imaging enabled to greatly reduce this effect. Automatic collection of the slices while imaging the coronal planes avoided extra collection time, and utilizing water flow to collect the slices was suitable for maintaining slice integrity and achieving a high rate of success for collection. Vibrating sectioning of the formaldehyde-fixed and agarose-embedded mouse brains had minimal detrimental effects on brain morphology and the corresponding datasets were considered to be free of distortions^40^. Reliable acquisition of smooth slices is critical for massive and systematic molecular classification of neural circuits. Our design of vibrating sectioning minimized vertical displacement to achieve high-precision sectioning. In addition, electromagnetic driving, rather than mechanical driving^42,43^, provided sustained high stability because there was no mechanical wear in system running. These designs enabled us to image and examine a single whole brain within approximately 10 hours and provide sequential, smooth and entire slices for subsequent experiments.

Recently, chemical clearing has attracted significant attention because this approach translates section immunostaining assays to whole-mount tissues^5–13^. However, non-area-selective whole-mount labelling leads to redundant immunostaining, and antibody penetration into large tissues remains a significant challenge. To date, most chemical clearing methods have presented immunostained images of millimetre-scale tissue blocks, and only a few methods have demonstrated whole-brain labelling of limited antibodies^5,8,12^. There was doubt that the immunostaining method would be effective for deep brain tissue in whole-brain samples owing to lack of demonstration. In addition, it must be noted that imaging deep tissue at high resolution in the whole brain using light-sheet microscopy remains difficult because of the technical limitations of residual refractive index mismatches; moreover, this blurry effect is exacerbated when the sample size increases. In our pipeline, the imaging-sectioning-collecting circle and subsequent slice staining successfully avoided uneven imaging and staining in the whole brain. Because we employed traditional slice staining, there were no limitations on antibody selection. In addition, targeted staining and analysis strategy prevented redundant labour, dramatically reduced cost compared with whole-mount immunostaining and markedly increased the phenotyping efficiency for specific neural circuits. Studying the primate brain has become increasingly important for understanding the structures and functions of the human brain. Mapping and phenotyping the primate brain provides challenges both for chemical clearing- or mechanical sectioning-based technologies. We feel that imaging cleared samples with mechanical sectioning-based technologies is promising. With the increase in sample size, the uniformity of clearing and antibody penetration decrease. Meanwhile, deterioration of the light sheet in large tissue leads to a resolution decrease. Recently, Economo *et al*.^39^ reported a combination of chemical clearing and vibrating sectioning-based imaging, and these authors demonstrated that cleared tissue could endure mechanical sectioning. However, the data acquisition speed of mechanical sectioning-based technologies is limited by sectioning thickness. Chemical clearing enables mechanical sectioning-based technologies to achieve deeper Z imaging ranges and thicker sectioning. Our method, which integrates chemical clearing, can achieve efficient automation and industrialization of cell type analyses in specific circuits with high resolution and throughput across species for the study of health and disease.

## Methods

### Animals

C57BL/6 J mice, *Thy1*-eGFP M-line transgenic mice, *Thy1*-eYFP H-line transgenic mice and *Ai14* Cre-reporter mice (Jackson laboratory, USA) were used in this study. Mice were housed on a 12-hour light/dark cycle with food and water provided *ad libitum*. The animal experiments were approved by the Institutional Animal Ethics Committee of Huazhong University of Science and Technology and all experiments were performed in accordance with relevant guidelines and regulations.

### Virus injections

Experiments in Figs 3 and 4 were performed in *Ai14* Cre-reporter mice that were 3–6 months old. The mice were anaesthetized with a 1% solution of sodium pentobarbital via intraperitoneal injection. The stereotaxic coordinates for MOp, SSp, VISp were based on the Mouse Brain in Stereotaxic Coordinates Atlas^44^. Using a pressure injector (Nanoject II, Drummond Scientific, USA), 300 nl of *CAV-Cre* (3 × 10^12^ viral particles per ml, Montpellier vectorology, France) was injected into the MOp, SSp or VISp of each *Ai14* mouse (1.65 mm A-P, 1.70 mm M-L and 1.25 mm D-V in MOp; 0.10 mm A-P, 2.30 mm M-L and 1.30 mm D-V in SSp; −3.25 mm A-P, 2.90 mm M-L and 1.30 mm D-V in VISp). After surgery, the animals were returned to standard living conditions for two weeks until they were sacrificed for brain sample preparation. Cre recombinase was delivered at the injection site by CAV, which efficiently transduced axon terminals. Then, the stop cassette of *Ai14* was removed by Cre recombinase, and tdTomato fluorescent protein was expressed in the input neurons.

### Tissue preparation

All histological procedures were as conducted as follows. Briefly, the mice were anaesthetized with a 1% solution of sodium pentobarbital and subsequently intracardially perfused with 0.01 M phosphate buffered saline (PBS, Sigma-Aldrich, USA), which was followed by 4% paraformaldehyde (PFA, Sigma-Aldrich, USA) in 0.01 M PBS. Then, the brains were excised and post-fixed in 4% PFA at 4 °C for 24 hours. After fixation, each intact brain was rinsed overnight at 4 °C in 0.01 M PBS and prepared for embedding. Oxidized agarose was made according to the following steps^45,46^. Agarose type I-B (Sigma-Aldrich, USA) was added to 10 mM sodium periodate (NaIO_4_, Sigma-Aldrich, USA) solution and stirred for 2~3 hours at room temperature. The oxidized product was repeatedly washed and resuspended in PBS to bring the final concentration to 5%. The brains were pat-dried and embedded in melted oxidized agarose using a silicone mould. There were several cuboid-shaped grooves in the mould for brain embedding and gridlines for correcting the brain orientation. The mould and brains were placed in a 55 °C water bath for 0.5 hours until the surfaces of the brains were fully coated with agarose. During the water bath, the orientation of the brains could be easily adjusted. Then, the mould and brains were left at room temperature for 0.5 hours to allow the agarose to solidify. After that, the brains were separated from the mould and stored in PBS at 4 °C before imaging.

### Instrument

The rapid whole-brain optical tomography system consists of three functional parts: SIM, vibratome and slice-collection device. The configuration of SIM was same as a previous DMD-based SIM, but was mainly used for rapid interval-sampling imaging with a thicker optical sectioning thickness, rather than high resolution continuous imaging^21^. The SIM enabled dual-channel optical sectioning with a 20×/1.0 NA objective (XLUMPLFLN 20XW, Olympus, Japan), two sCMOS cameras (ORCA-Flash 4.0, Hamamatsu, Japan) and filters (FF01-468/553, FF493/574-Di01, FF01-512/630, FF562-Di03, Semrock, USA). The optical sectioning thickness (Supplementary Fig. 2) was flexible by adjusting the pattern period of DMD (XD-ED01N, X-digit, China). The pixel size of DMD was 13.68 µm × 13.68 µm, and the magnification between DMD and the focal plane of the objective was 16.7×. The typical-use pattern period was 18 micro-mirrors and corresponded to 14.8 µm at the focal plane. Owing to the limited size of DMD and uneven illumination, we only recorded a subarray of 1,600 × 1,600 pixels in sCMOS cameras for a total of 2,048 × 2,048 pixels, which represented approximately 61% of the effective area. We chose an axial response of 5.8 µm at 510 nm to balance the effective information and signal-to-noise ratio in whole-brain imaging. Somas and nerve fibers can be clearly distinguished at this axial resolution (Supplementary Fig. 2 and Supplementary Figs 6–9). Using higher frequency illumination pattern brings a higher axial resolution of 2.4 μm (Supplementary Fig. 2), but also lower signal-to-noise ratio and less effective information. In whole-brain imaging, the size of data is directly determined by sampling rate rather than axial resolution. For 50-μm-interval whole-brain imaging, each single coronal image is collected at an interval of 50 μm, and the size of datasets is the same despite of the axial resolution of 5.8 μm or 2.4 μm. For full-volume whole-brain imaging with axial resolution of 5.8 μm, 2,870 coronal sections were collected at z-steps of 5 µm in Supplementary Fig. 6. The whole brain can also be imaged at z-steps of 2 µm, which leads to 2.5 times as much data (~7170 coronal images) as the former.We developed a high-precision leafspring electromagnetic-based vibratome, as shown in Supplementary Fig. 3a, to implement excellent and stable vibrating sectioning. The leafsprings (93.0 × 49.0 × 1.6 mm) were made of spring steel. This double-leafspring structure was compliant in the Y direction but was extremely stiff in the Z direction to resist parasitic vertical movement. Two neodymium magnet blocks (30 × 20 × 10 mm, grade N52) and two steel pole shoes were used to generate an even magnetic field. The rectangular coils included ~1,100 windings of insulated copper wire (0.35 mm diameter) wrapped around an aluminium coil bobbin. The coils were mounted on the base, and one side of the coils was in the magnetic field. To maximize the driving efficiency, the modulation frequency of AC voltage was the same with the resonance frequency of the vibrating structure. A high-precision adjustment screw (254 thread per inch; AJS254, Newport, USA) was used to adjust the rolling angle of the blade. The knife vibrated at the natural frequency (80.5 Hz) of the vibratome. We used a ceramic blade (7550-1-C, Electron Microscopy Sciences, USA) for sectioning because this material is much more rigid and durable than a stainless steel blade. We developed a homemade photo-electric detection device to measure the Z displacement of the blade (Supplementary Fig. 3b). The device consisted of an infrared emitting diode (IRED), a photodiode (PD) and an amplifying circuit. The signal intensity of the PD changed with the Z position of the blade. Detection sensitivity was approximately 100 mV/µm.

A micro gear pump (MG1012A, Pascal micropump, China) was used to generate water flows of different directions in the silicone pipe (8 mm diameter). The T-container, valves and distributer were mounted on a 3-axis robot arm (X: T6L, Y: T5L, Z: T4L, Yamaha, Japan). The repeatability of the X/Y/Z axis was ±0.02 mm, which guaranteed accurate alignment between the distributer and each well of the multi-well plates.

A 3D stage (X: ABL2000, Y: ANT130-L, Z: AVL125, Aerotech, USA; Resolution: 10 nm, 1 nm, 4.5 nm, respectively) moved the sample to extend the FOV and move it towards the oscillatory blade to generate slices. The travel ranges of the X/Y/Z axis were 200, 60 and 25 mm, respectively, which were large enough for a centimetre-sized mouse brain. The repeatability of the X/Y/Z axis was ±200 nm, ±75 nm and ±300 nm, respectively, which guaranteed precise registration of images between different FOV and sections. The vibratome, valves and pump were controlled by the software through the digital I/O port of the controller of the 3D stage.

### System workflow

The system worked automatically as follows (Supplementary Fig. 1). (i) The sample was moved to the imaging part, and the whole image of the superficial tissue of the sample was acquired by mosaic scanning. (ii) The sample was sequentially moved to the sectioning part, and the imaged tissues were removed by the vibratome. The blade holder was specially designed to connect the sectioning and collecting parts through a silicon pipe. Two pinch valves and a pump were employed to control water flow. In this step, Valve-1 was open and Valve-2 was shut. A small water flow along the direction of Flow-1 avoided the sectioned tissue that floated away during sectioning. (iii) Once sectioning was completed, the water flow was immediately increased to push the slice into silicon pipe-1 via the through-hole of the blade holder and moved into the T-container. A strainer was set before the pump to avoid the slice that was moved into the pump. Then, Valve-1 was shut, Valve-2 was opened, and the pump reversed the direction of the water flow. The water flowed along the direction of Flow-2 to push the slice into a corresponding well of the multi-well plates through the distributer. Strainers were fixed on each well bottom of the multi-well plates to keep the slices in the well and drain away water. We put the multi-well plates in a collecting tank to always keep the slice in water. After the slices were added to the tank, a 3-axis robot arm moved the distributer away, and a strong water flow washed potential residues on the internal wall of the T-container and pipe-2 away. Then, the distributer was moved back to the next well to wait for the next operation.

### Instrument operation

Before imaging, the vibration amplitude of the blade was set to 1 mm by adjusting the voltage amplitude, and the Z displacement of the blade was controlled in 1 µm by adjusting the screw. The sectioning speed was set to 0.5 mm/s. Then, the sample block was fixed in the bottom of a water tank filled with PBS. The leading edge of the specimen block was aligned to be parallel to the cutting edge of the blade. Then, the top of the specimen block was brought up to a level similar to the cutting edge by adjusting the z-axis of the 3D stage. Redundant parts of the agarose block were trimmed off, and the specimen was moved to the starting point of imaging. Automated imaging, sectioning and slice collection were implemented with custom software written in C++. For 50-μm-interval whole-brain imaging (~280 sections; experiments in Fig. 2), the data collection time included ~7.8 hours for imaging and ~3.2 hours for sectioning. For 40-μm-interval whole-brain imaging (~360 sections; experiments in Fig. 3), the data collection time included ~9.6 hours for imaging and ~3.9 hours for sectioning.

### Sectioning quality of brain tissue

For sectioning-based whole-brain imaging technologies, high-quality sectioning allows an imaging plane to be maintained close to the sample surface for reducing additional optical scattering in the tissue. Moreover, stable and reliable long-term data acquisition requires high durability for sectioning. Because the brain tissue is too soft to be directly measured by a stylus profiler, we used an alternative approach by imaging the fresh cutting plane of the brain tissue to evaluate the sectioning quality. Rugged topography led to optical sectioning at the top surface to miss partial signals below the imaging plane, as shown in the inset of Supplementary Fig. 5a. As mentioned above, the flat cutting face allowed us to avoid this type of problem, as shown in the inset of Supplementary Fig. 5b.

To present this influence of sectioning quality on imaging, we imaged the real time-stained blockface of two 2 M C57BL/6 J mouse brains using our non-optimized and optimized system, respectively. Imaging of an entire coronal section of mouse brain took about 1 to 4 minutes. And the penetration depth of PI into agarose-embedded brain tissue was less than 10 µm during a 5-minute immersion in PBS containing PI (3 μM) (Supplementary Fig. 4). This real time-staining of superficial tissue enabled us to evaluate sectioning quality using optical sectioning imaging. The vibrating sectioning thickness was 25 µm. Two whole-brain datasets of approximately 560 coronal images were acquired under the same imaging conditions. After system optimization, good sectioning quality of the system led to entire uniform signal distribution without any missing parts (Fig. 1d and Supplementary Fig. 5b), much better than the unoptimized results (Fig. 1c and Supplementary Fig. 5a).

### Staining and immunostaining of collected brain slices

For acquiring the outline of the sequential collected slices (Experiments in Fig. 2), the DAPI at a dilution of 5 µg/ml was used to stain the slices. For characterizing the molecular phenotypes in LC and PAL (Experiments in Fig. 4), the corresponding slices through LC and PAL were selected according to the whole-brain datasets and transferred to 24-well plates. The slices were washed using PBS for 3 × 10 minutes, permeabilized using PBST (0.3% Triton-X100 in PBS) for 1 hour and then incubated with blocking solution (5% Bovine Serum Albumin (BSA) in PBST) for 1 hour followed by incubation with primary antibodies overnight at 4 °C. The primary antibodies were as follows: all slices for LC immunostaining included rabbit anti-TH (1:1,000 dilution, T8700; Sigma-Aldrich, USA); a set of slices for PAL dual-immunostaining included rabbit anti-ChAT (1:600 dilution, AB143; Millipore, German) and mouse anti-PV (1:1,000 dilution, MAB1572; Millipore, German); and another set of slices for PAL dual-immunostaining included goat anti-ChAT (1:1,000 dilution, PA5-18518; Invitrogen, USA) and rabbit anti-CB (1:800 dilution, ab11426; Abcam, UK). After washing the slices 3 × 10 minutes in PBS, the appropriate secondary antibodies were applied for 2 hours at room temperature. The secondary antibodies used were as follows: Alexa Fluor 647 donkey anti-goat IgG, Alexa Fluor 488 donkey anti-rabbit IgG, Alexa Fluor 488 goat anti-rabbit IgG, and Alexa Fluor 405 goat anti-mouse IgG, (all dilution at 1:1,000, A-21447, A-21206, A-11034, A-31553, respectively; Invitrogen, USA). Later, the slices were washed once more with PBS prior to mounting and were coverslipped with Fluorogel. The fluorescence images were acquired with a Zeiss 710 LSM confocal microscope. For quantifications of the molecular phenotypes of LC and PAL, we manually counted the stained cells using ImageJ (NIH).

### Image pre-processing and visualization

The original mosaic images of each coronal section were saved in a lossless TIFF format. The neighbouring overlap of the images was removed and stitched to an entire section. The uneven lateral illumination in each section was corrected according to correction coefficients determined by mean intensity along each direction and fitting corresponding polynomial curves^21^. All sections were saved at an 8-bit depth in an LZW compression TIFF format after image pre-processing. We visualized the whole brain datasets using Amira software (v 5.2.2, FEI, France) to generate figures of maximum intensity projection, volume and surface rendering. Our system acquired each image of superficial part of the tissue block before sectioning and collecting. Thanks to the specific workflow and the stability of the system, the integrity of the whole-brain imaging dataset was guaranteed, although a few sample slices may be lost. Thus no specific interpolation algorithms or templates were used for rendering.

## Electronic supplementary material

Supplementary information 

Supplementary Movie 1
