# Supplementary material for: A platform for efficient identification of molecular phenotypes of brain-wide neural circuits
Source: Sci Rep. 2017 Oct 24;7:13891. doi: 10.1038/s41598-017-14360-6 (PMC5654830; doi:10.1038/s41598-017-14360-6)
Supplement: Supplementary file 1 — Supplementary information [file 41598_2017_14360_MOESM1_ESM.doc]

**A platform for efficient identification of molecular phenotypes of brain-wide neural circuits**

Tao Jiang^1,2*^, Ben Long^1,2*^, Hui Gong^1,2^, Tonghui Xu^1,2^, Xiangning Li^1,2^, Zhuonan Duan^1,2^, Anan Li^1,2^, Lei Deng^1,2^, Qiuyuan Zhong^1,2^, Xue Peng^1,2^, Jing Yuan^1,2^

^1^Britton Chance Center for Biomedical Photonics, Huazhong University of Science and Technology-Wuhan National Laboratory for Optoelectronics, Wuhan 430074, China

^2^Key Laboratory of Biomedical Photonics (Huazhong University of Science and Technology), Ministry of Education, Department of Biomedical Engineering, Huazhong University of Science and Technology, Wuhan 430074, China

^*^ These authors contributed equally to this work. Correspondence and requests for materials should be addressed to J.Y. (email: yuanj@mail.hust.edu.cn).

**Supplementary figures and videos list**

| **Supplementary Figure 1** | Workflow of the Rapid whole-brain optical tomography system. |
| --- | --- |
| **Supplementary Figure 2** | Optical sectioning of the system. |
| **Supplementary Figure 3** | Leafspring electromagnetic-based vibratome. |
| **Supplementary Figure 4** | Real-time penetration of PI into agarose-embedded mouse brain tissue. |
| **Supplementary Figure 5** | Sectioning performance comparison of the system before and after optimization. |
| **Supplementary Figure 6** | Full-volume imaging dataset of a *Thy1*-eGFP M-line mouse brain. |
| **Supplementary Figure 7** | Coronal sections showing the distribution of inputs to MOp from selected brain regions. |
| **Supplementary Figure 8** | Coronal sections showing the distribution of inputs to SSp from selected brain regions. |
| **Supplementary Figure 9** | Coronal sections showing the distribution of inputs to VISp from selected brain regions. |
| Supplementary Table 1 | Success rate of slice collection. |
| **Supplementary Movie 1** | Closeup of the system at a vertical view at runtime. |

**Supplementary figures**

**
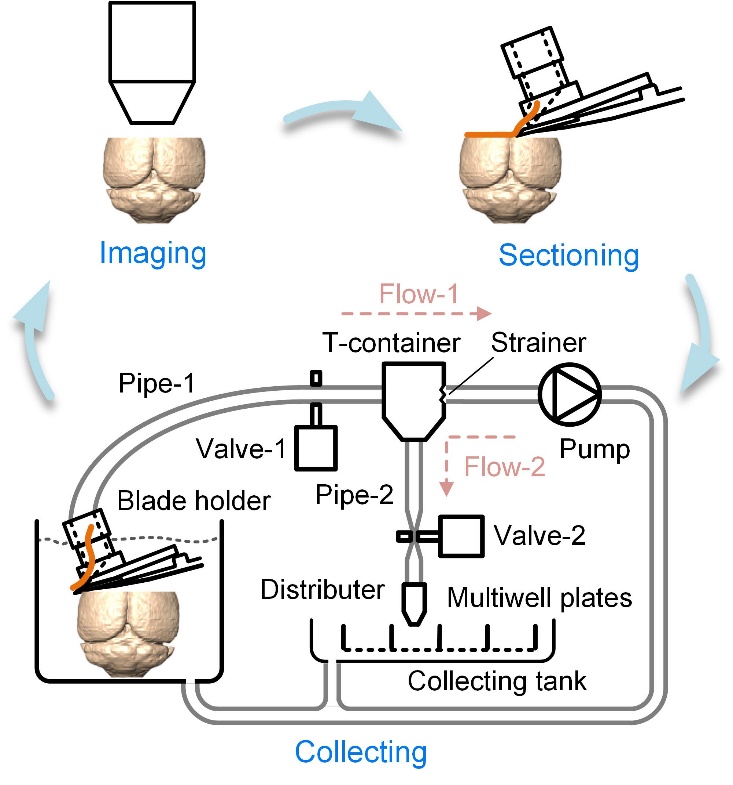
**

**Supplementary Figure 1. Workflow of the Rapid whole-brain optical tomography system.**

Flow-1 and Flow-2 represent different directions of water flow in the slice-collection device. Detail description of the system workflow is in materials and methods.


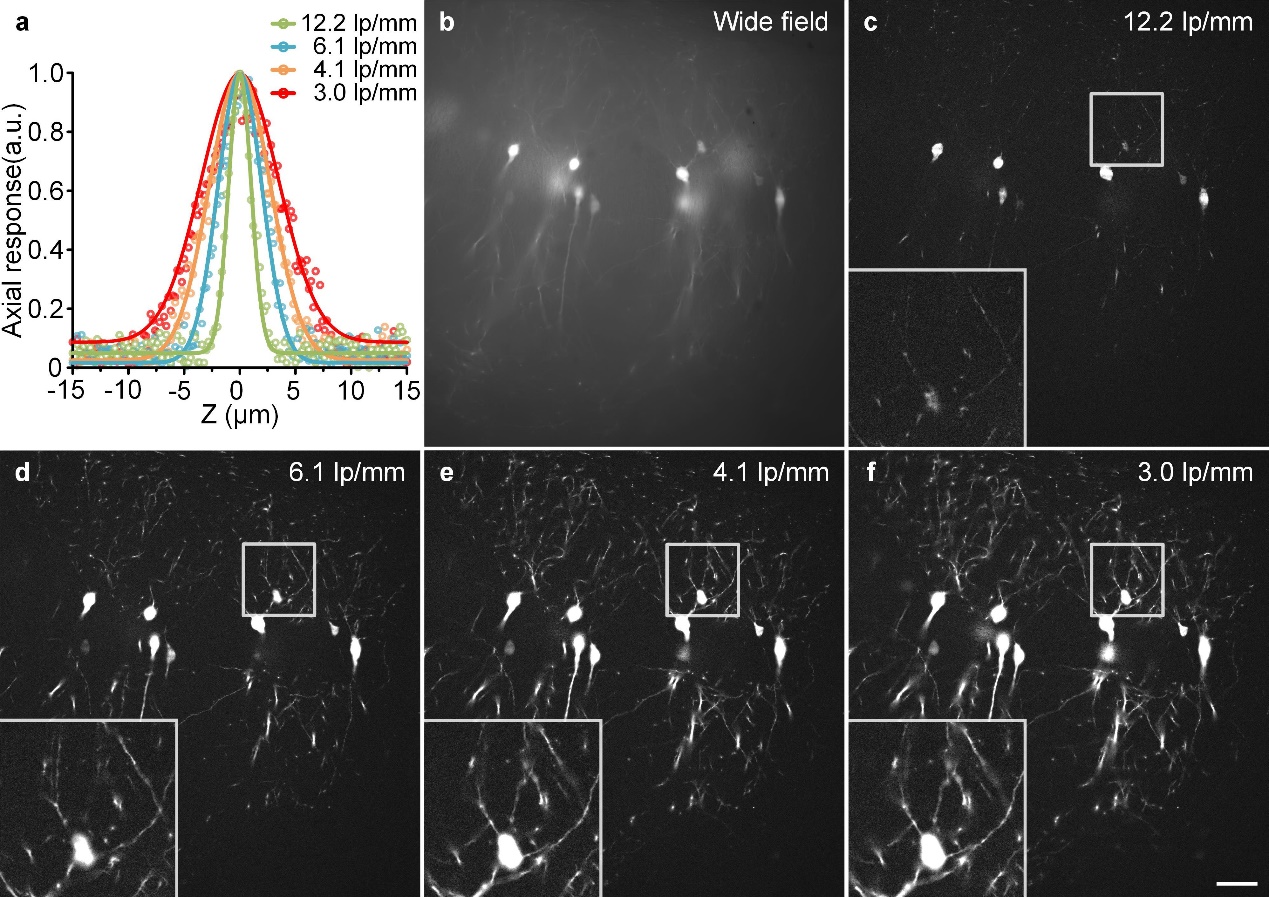


Supplementary Figure 2. Optical sectioning of the system.

(a) Axial response vs. illumination pattern frequency on the DMD. We modulated the pattern with periods of 6, 12, 18 and 24 micro-mirrors and imaged a thin fluorescence film (fluorescein sodium) as an axial response^47, 48^. The theoretical full width at half maximum (FWHM) values were 2.0, 3.9, 5.8 and 7.8 µm at 510 nm, respectively, and the corresponding measured FWHM values were 2.4, 4.7, 6.5 and 8.4 µm, respectively. (b) Wide-field images of an agarose-embedded *Thy1*-eYFP H-line mouse brain block. (c-f) Optical sectioning images with different illumination patterns of the same FOV at the same exposure time. Scale bar: 50 µm.


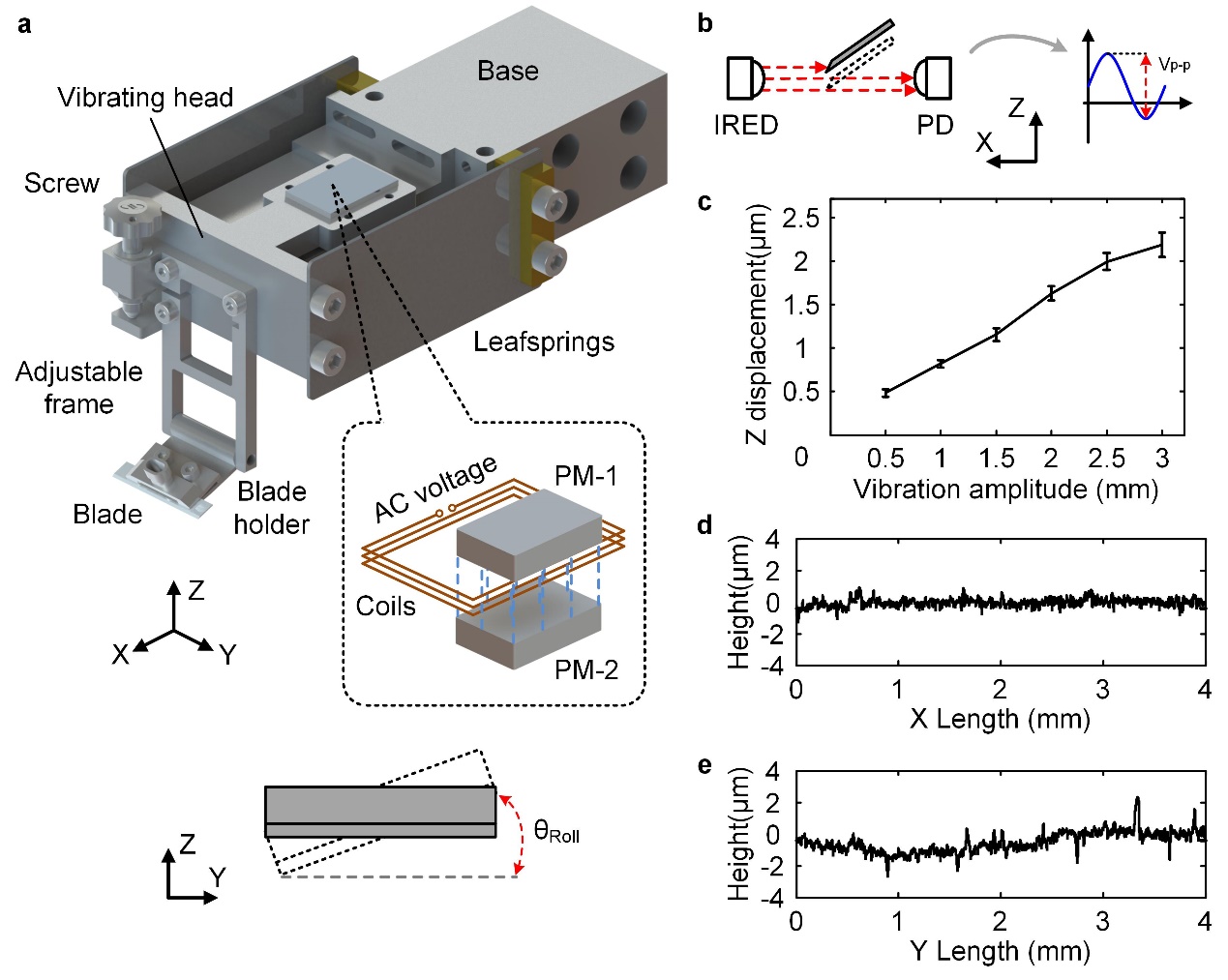


**Supplementary Figure 3. Leafspring electromagnetic-based vibratome.**

(**a**) Construction of the leafspring electromagnetic-based vibratome. PM: permanent magnet. (**b**) Detection device for Z displacement measurements. IRED, infrared emitting diode. PD, photodiode. (**c**) The Z displacements (peak-to-peak amplitude) of the blade at different vibration amplitudes. Data are shown as the mean ± SD, n = 5. (**d**) and (**e**) Typical surface flatness of the cutting face of the agarose in the X and Y directions, respectively. The sectioning amplitude and speed of the vibratome were 1.0 mm and 0.5 mm/s, respectively. The Surface flatness was measured by a stylus profiler (Dektak XT, Bruker, Germany).


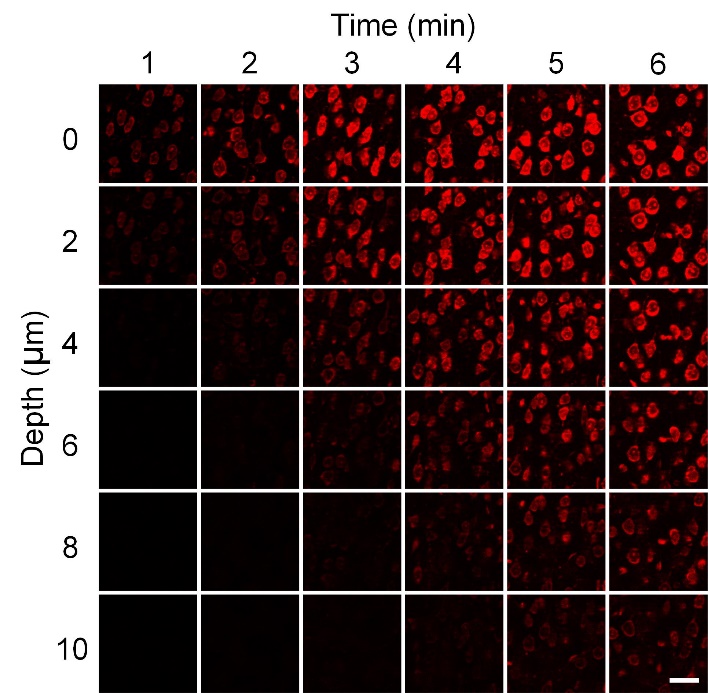


**Supplementary Figure 4.** **Real-time penetration of PI into agarose-embedded mouse brain tissue.**

Once the brain of a 2M C57BL/6 mouse was sectioned, PI (3 μM) molecules penetrated immediately. An image stack (z step of 2 µm and voxel size of 0.32 × 0.32 × 2.0 µm) was acquired after a penetration time of 1 minute. Then, the tissue was sectioned at a thickness of 20 µm, and the above experiments were repeated for penetration times of 2, 3, 4, 5 and 6 minutes. The scale bar is 30 µm.

**
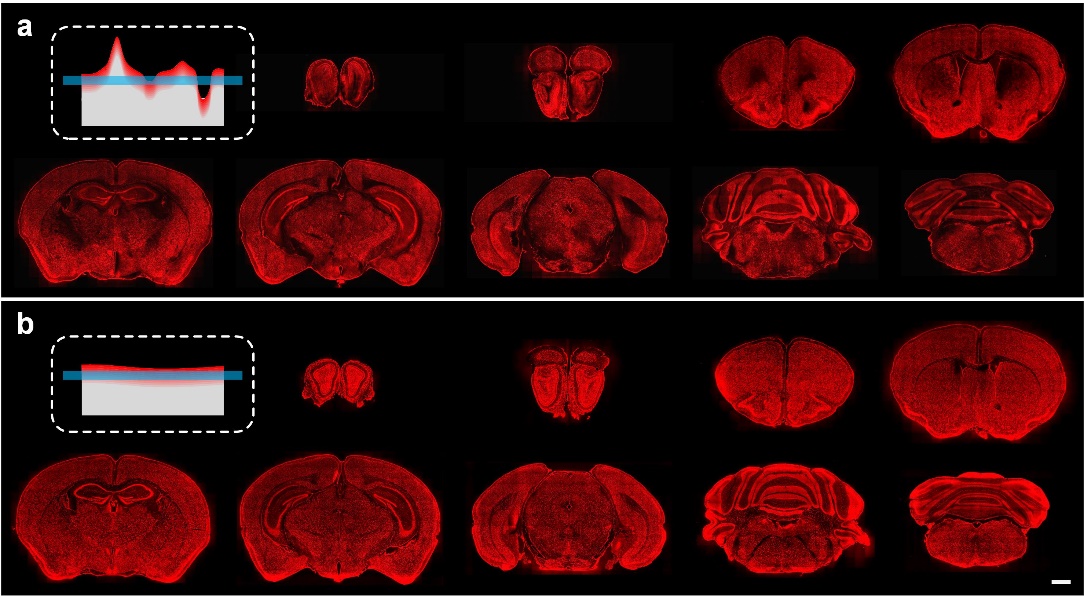
**

**Supplementary Figure 5. Sectioning performance comparison of the system before and after optimization.**

Two series of evenly spaced coronal images of real time-PI-stained 2M C57BL/6J mouse brains acquired by the system before and after optimization are shown in (**a**) and (**b**), respectively. The data was from the same datasets of **Figs.1c** and **d**, respectively. Scale bar: 1 mm. The insets in (**a**) and (**b**) are schematic diagrams of imaging unflat- and flat-surface tissue, respectively. White represents the tissue sample, red represents PI-staining in the superficial region of tissue, and blue represents the optical sectioning imaging plane.


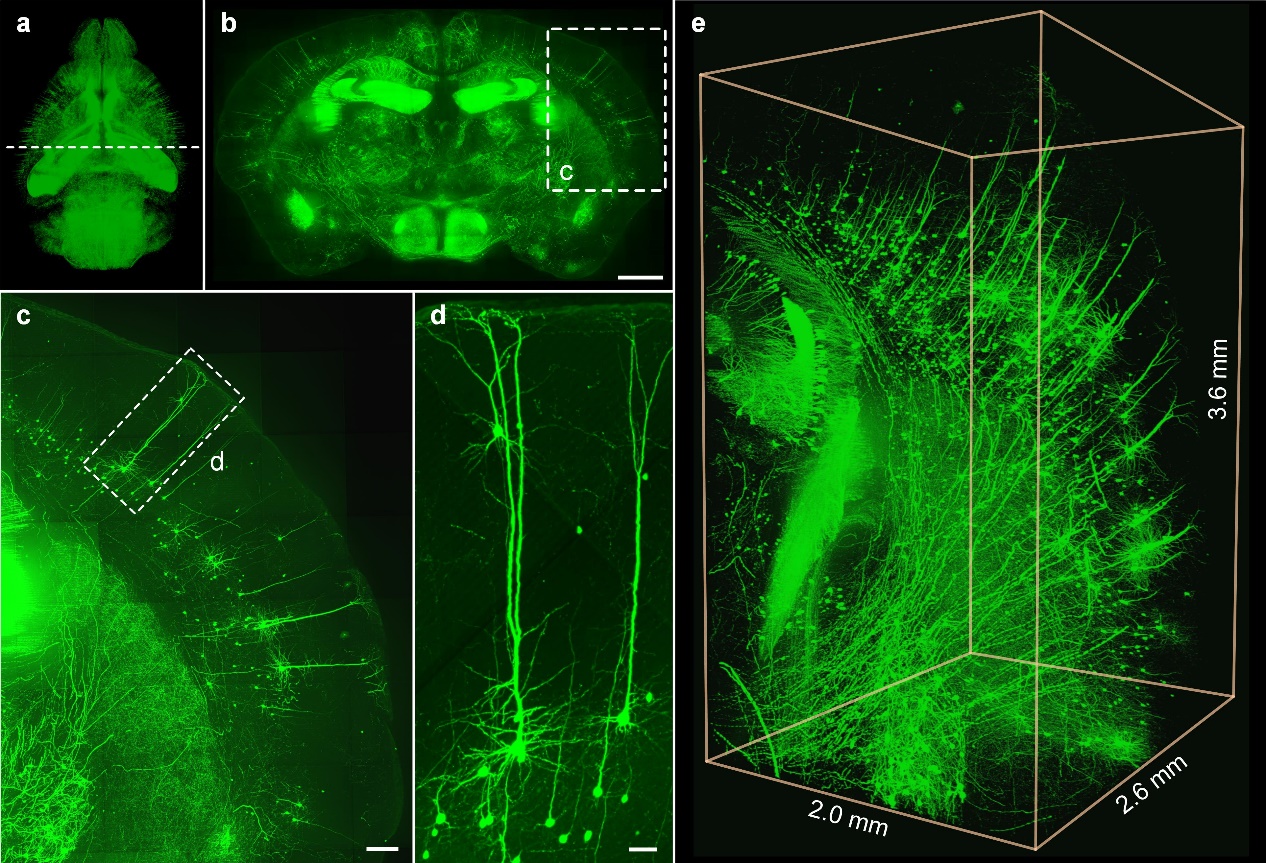


Supplementary Figure 6. Full-volume imaging dataset of a *Thy1*-eGFP M-line mouse brain.

(a) 3D volume rendering of the whole-brain dataset. (b) Maximum intensity projection over a 200-μm-thick data volume. The coronal image refers to the dashed line shown in (a). (c) Enlarged view of the region defined by the dashed rectangle in (b). (d) Enlarged view of typical layer V pyramidal neurons in the dashed rectangle in (c). (e) High-resolution 3D volume rendering of local brain regions corresponding to the rectangle in (b). Volume size: 2.6 × 3.6 × 2.0 mm. Scale bars: 1 mm in (b), 200 µm in (c) and 50 µm in (d).


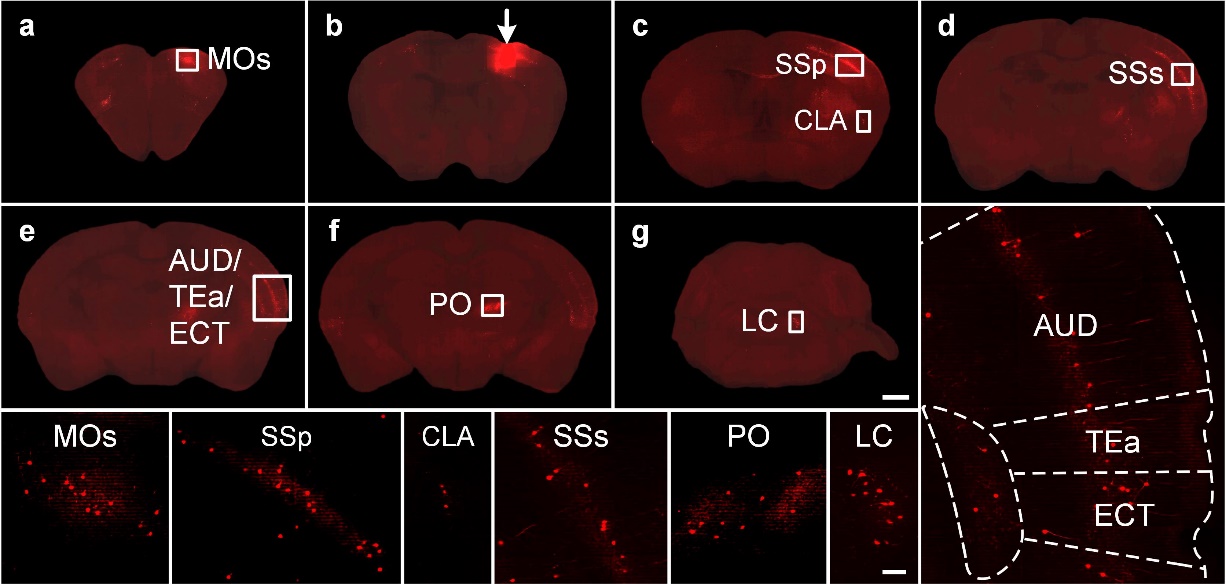


**Supplementary Figure 7. Coronal sections showing the distribution of inputs to MOp from selected brain regions.**

The proportion of the inputs higher than or equal to 2.0%. The scale bar is 1 mm. The insets show corresponding partial magnified images in (**a**) - (**g**). The scale bar is 100 μm. The white arrow indicates the injection site.


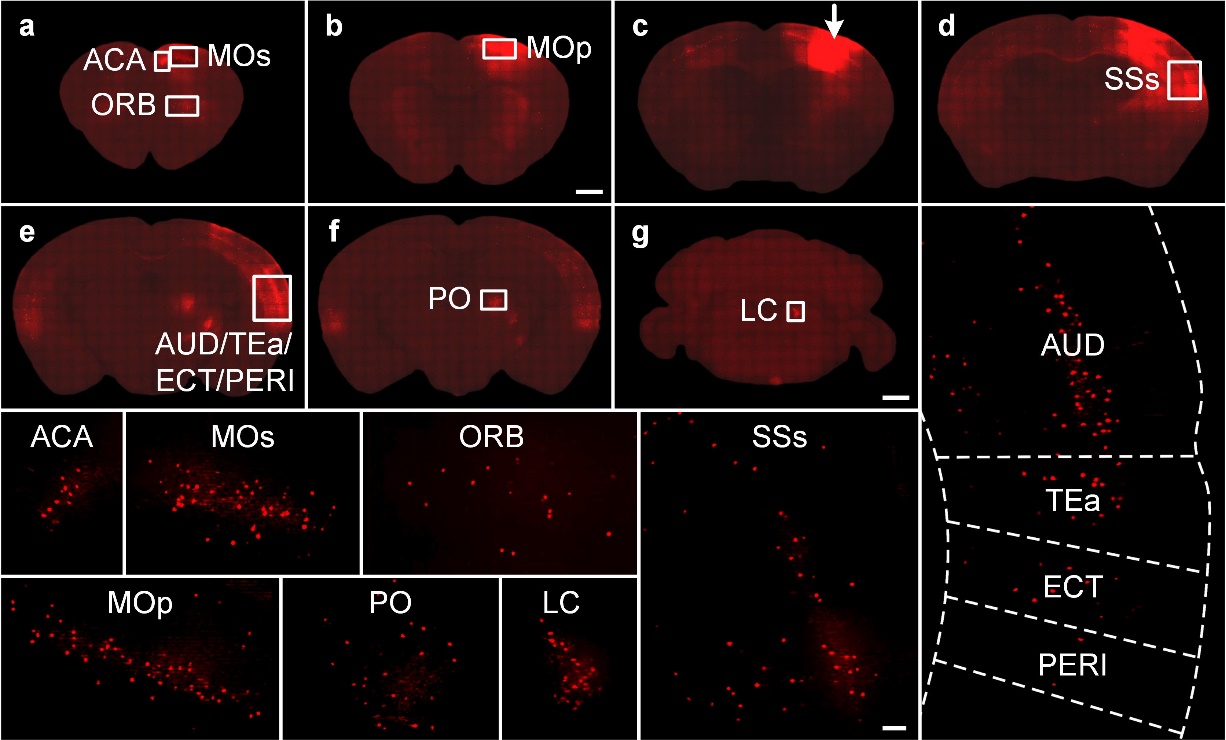


**Supplementary Figure 8. Coronal sections showing the distribution of inputs to SSp from selected brain regions.**

The proportion of the inputs was higher than or equal to 2.0%. The scale bar is 1 mm. The insets show the corresponding partial magnified images in (**a**) - (**g**). The scale bar is 100 μm. The white arrow indicates the injection site.


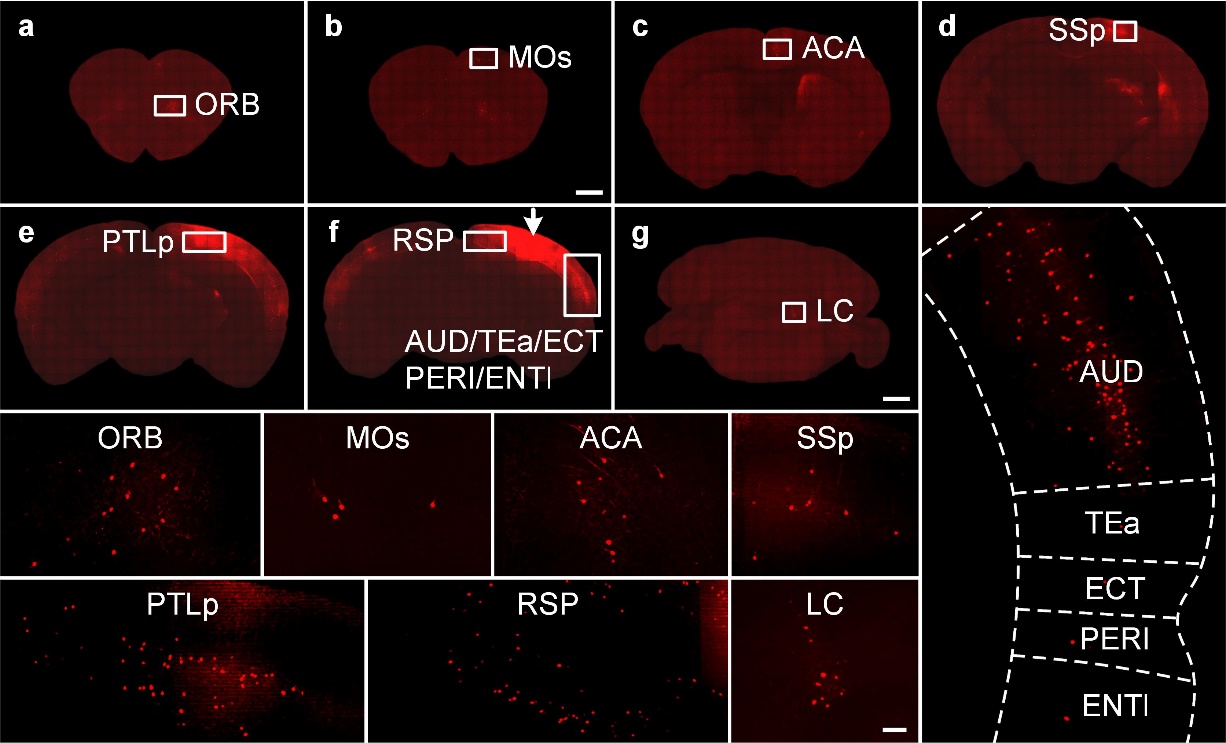


**Supplementary Figure 9. Coronal sections showing the distribution of inputs to VISp from selected brain regions.**

The proportion of the inputs was higher than or equal to 2.0%. The scale bar is 1 mm. The insets show the corresponding partially magnified images in (**a**) - (**g**). The scale bar is 100 μm. The white arrow indicates the injection site.

**Supplementary Table**

Supplementary Table 1. Success rate of slice collection.

| Sample | Total slices | Missing slices | Partially lost slices | Collected intact slices | Success rate |
| --- | --- | --- | --- | --- | --- |
| #1 | 272 | 2 | 4 | 266 | 97.8% |
| #2 | 288 | 1 | 8 | 279 | 96.8% |
| #3 | 287 | 4 | 4 | 279 | 97.2% |
| #4 | 283 | 0 | 6 | 277 | 97.9% |
| Average |  |  |  |  | 97.4% |

**Supplementary Movie caption**

**Supplementary Movie 1**. Closeup of the system at a vertical view at runtime. White arrow indicates the movement of the slice in the silicon pipe.
